# Multimodal hand gesture recognition using single IMU and acoustic measurements at wrist

**DOI:** 10.1371/journal.pone.0227039

**Published:** 2020-01-13

**Authors:** Nabeel Siddiqui, Rosa H. M. Chan

**Affiliations:** Department of Electrical Engineering, City University of Hong Kong, Tat Chee Avenue, Kowloon, Hong Kong, China; University of Houston, UNITED STATES

## Abstract

To facilitate hand gesture recognition, we investigated the use of acoustic signals with an accelerometer and gyroscope at the human wrist. As a proof-of-concept, the prototype consisted of 10 microphone units in contact with the skin placed around the wrist along with an inertial measurement unit (IMU). The gesture recognition performance was evaluated through the identification of 13 gestures used in daily life. The optimal area for acoustic sensor placement at the wrist was examined using the minimum redundancy and maximum relevance feature selection algorithm. We recruited 10 subjects to perform over 10 trials for each set of hand gestures. The accuracy was 75% for a general model with the top 25 features selected, and the intra-subject average classification accuracy was over 80% with the same features using one microphone unit at the mid-anterior wrist and an IMU. These results indicate that acoustic signatures from the human wrist can aid IMU sensing for hand gesture recognition, and the selection of a few common features for all subjects could help with building a general model. The proposed multimodal framework helps address the single IMU sensing bottleneck for hand gestures during arm movement and/or locomotion.

## Introduction

Human fingers are one of the main means of interaction with the world and are an essential body part in the study of gesture recognition technologies in the field of human–computer interaction (HCI). Gesture recognition technology allows humans to interact with a remote system without physical contact. Many types of sensors have been used in wireless systems for gesture recognition including cameras, sensor gloves, and muscle-based gadgets [1]. The conventional data glove records finger orientation by measuring flexion, vision-based devices commonly use a camera with a depth-based sensor, and muscle activity-based apparatuses record muscular contractions, known as surface electromyography (sEMG). The recordings of these time-series measurements of hand gestures are linked to some instruction in a computer. Despite the high accuracy (>90%) of each of these modalities, there are some limitations in their long-term usage [2,3]. For instance, wearing the data glove for longer durations is inconvenient, and having a camera positioned throughout daily life to track fingers is impractical. Nonetheless, we anticipate a practical gesture recognition system could be integrated with a smartwatch, the most widely used wearable wrist gadget. Finger movements are linked with the physical operation of tendons, bones, and ligaments at the wrist [4–7]. Some studies have shown that an optical sensor [4], accelerometer and gyroscope [5,6], an array of barometric sensors [8], and a combination of sEMG, accelerometer, and gyroscope [9] around the wrist could be used for hand gesture recognition at the wrist. An inertial measurement unit (IMU), containing an accelerometer and a gyroscope, is a lightweight tiny chip that can be easily placed over the wrist [6]. However, many studies have shown that an IMU sensor placed over the wrist in skin contact could be insufficient as a sole modality for hand gesture recognition, especially during arm movements [9]. If the IMU is not placed in skin contact, it may not detect information that could be recorded from the vibration over the skin while making a gesture. Many researchers have used IMU with other modalities to increase the accuracy of gesture recognition, e.g., IMU with sEMG [9].

In this study, we aimed to use pressure-based sensing with IMU at the wrist using pressure-based sensing units. Pressure-based transducers are more suitable where body movement is unavoidable [10]. Using microphones as pressure-based sensors, we proposed that relevant data could be collected at the wrist for hand gestures using microphones in our previous study [11]. This setup is also supported by another recent study [12]. The microphone signal, or acoustic measurement, is related to the vibrations generated over the skin due to the lateral movement of tissues inside the skin during any mechanical action. Microphones are used as pressure-based sensors in monitoring muscular data, where skin displacement causes a pressure change in the air chamber. Since the chamber is attached to the sound port of the microphone, the pressure change displaces the diaphragm of the microphone, which generates an electrical signal. This skin displacement is due to the low-frequency oscillations produced from muscle contractions [10,13,14]. Acoustic measurements taken from the muscle belly are commonly known as mechano-myography, sound myography, and acoustic myography [15–17].

**Fig 1:**
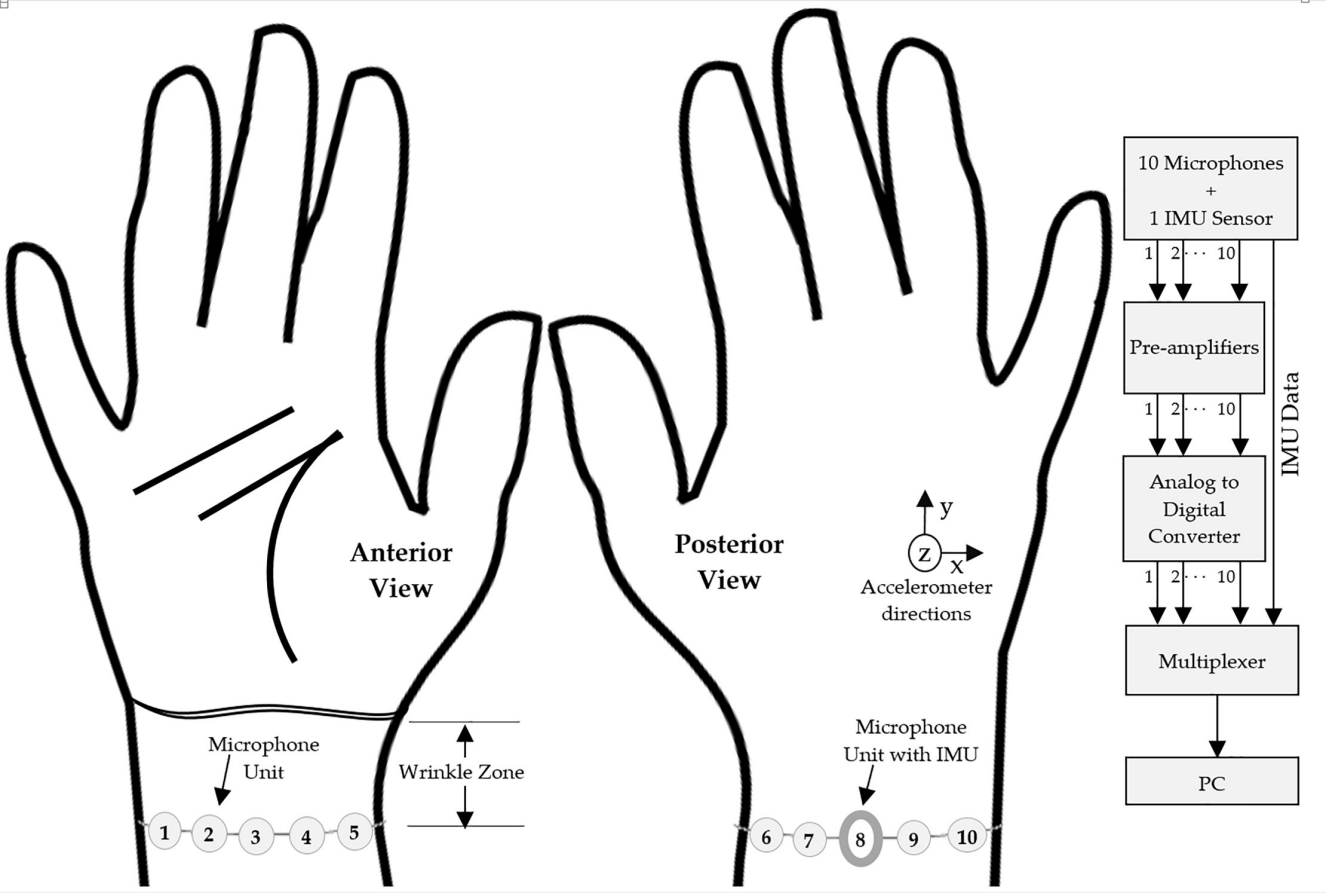
Illustration of hardware prototype. Sensor arrangement on the right hand’s anterior and posterior sides is shown at the left and the signal acquisition process is shown at the right.

Our approach of using multimodal sensors (microphones and IMU) at the wrist does not require specific muscle(s) for sensor placement, unlike sEMG [18]. However, specific area over the wrist which would provide significant information to support the use of acoustic sensors were yet to be explored. Therefore, a band of 10 acoustic sensors was created and spread around the wrist with an IMU attached to the band to record limb movement, as shown in Fig 1. Unlike some of the previous studies, the forearm is not fixed; therefore, the IMU is allowed to record every natural movement of the limb during gesture recording. Our main objective was to investigate if a subset of microphone units out of 10 microphone units could be used with an IMU to increase classification accuracy without confining natural limb movements.

First, we recorded 10 acoustic channels and 6 channels of IMU data (10 microphones, three-axis accelerometer, and three-axis gyroscope) from the wrists of 10 subjects. These subjects performed 10 trials for each of the 13 daily life gestures: hand lift, hands up, thumbs up/down, single/double tap, hand/finger swipe, okay sign, victory sign, and fist. Over 7000 features were then extracted from each channel, and the features most relevant to gesture recognition were selected using a mutual information-based algorithm. The results support a multimodal sensor approach using a single microphone unit placed at mid-anterior wrist along with an IMU. Using only two sensors (an IMU and a microphone unit at the mid-anterior wrist) with 25 features, the average accuracy for the general model was 75% for the given gesture set, whereas the average intra-subject classification accuracy exceeded 80% using the same features. These results can be achieved using either a support vector machine (SVM) or linear discriminant analysis (LDA) classifier. Another analysis was also performed using the data from IMU only, which showed that using a microphone with an IMU increased accuracy by around 5% compared with using IMU alone in the general model.

## Data acquisition system setup

**Fig 2:**
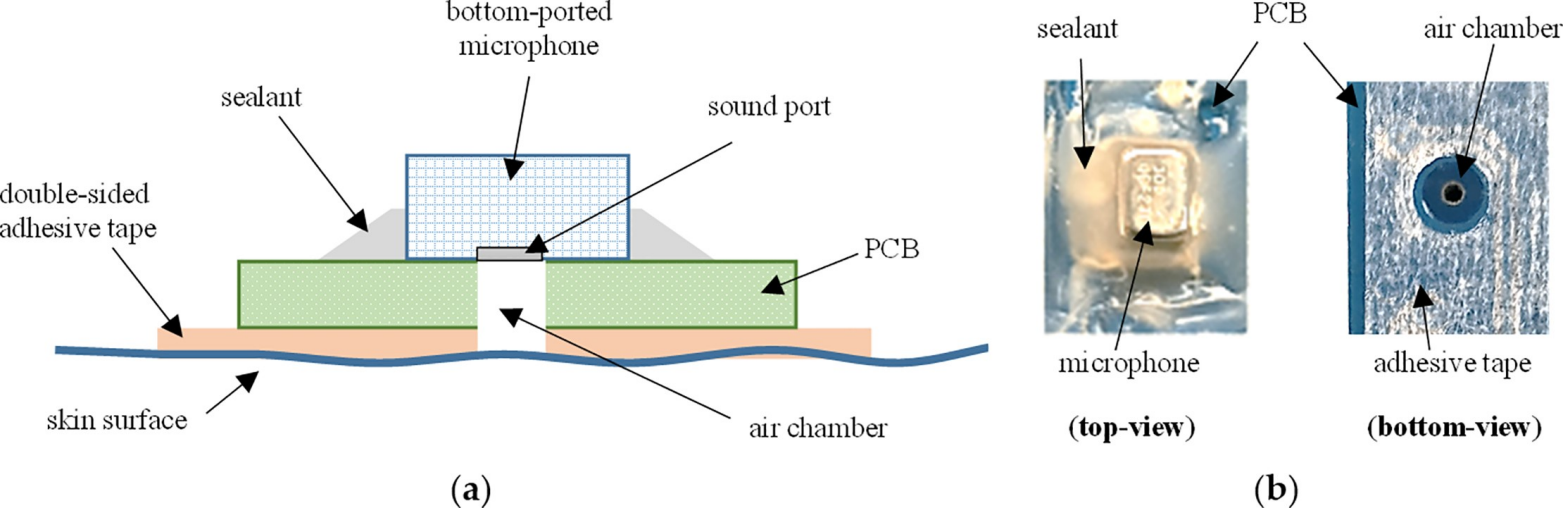
Microphone sensor unit placed on skin surface with double-sided adhesive tape. (a) Schematic cross-sectional side-view; (b) real-life shots.

Fig 1 illustrates the design of our hardware prototype, including the placement of all 10 microphone units. Each of these units had a bottom-ported microphone (f-3dB = 6 Hz; Model: ICS40300, InvenSense, San Jose, CA, USA) that was soldered to a custom designed circuit board (FR-4, 1.6 mm thickness). The cylindrical hole through the PCB acted as a hollow chamber between the microphone diaphragm and the skin surface. This setup is shown in Fig 2A and is similar to the one shown in Kim et al. [19]. An IMU (Model: LSM9DS0, STMicroelectronics, Geneva, Switzerland) was placed over microphone unit #8, which was positioned over the middle of exterior wrist, or, in other words, at the top of the entire sensor arrangement over the wrist, as illustrated in Fig 1. Our prototype utilized only one IMU to record limb movement and it was not in skin contact with the wrist. The procedure was adopted from Repnik et al. [20]. In a preliminary study, we used a single band of five microphones to validate the performance of hand gesture recognition from acoustic recordings [10]. In this study, we examined the multimodal functioning of our custom designed microphone units with an IMU. The distance between adjacent microphone units in a sensor band varies due to variation in the circumference of wrist amongst subjects, whereas the placement distance of sensor band from the hand over the wrist is discussed in detail in the next section.

As shown on the right side of Fig 1, the microphone units were connected to 10 pre-amplifier (preamp) units in parallel. Each preamp had an op-amp (Model: OPA344, Dallas, TX, USA) with a 67 dB gain arrangement and a lower cutoff frequency of 4 Hz. The pre-amplified acoustic signals and the signals from the IMU were fed to the recording computer via a data acquisition device (Model: Arduino DUE, Somerville, MA, USA). The sampling frequency of each acoustic channel was set to 200 Hz, because previous studies reported that the most significant bio-acoustic information occurs below 100 Hz [21]. The sampling rates for accelerometer and gyroscope were 100 Hz and 50 Hz respectively. Arduino was programmed at 200 Hz per channel sampling for recording every analog/digital signal. Since the sampling rates of accelerometer and gyroscope were different, therefore, these IMU signals were down-sampled in MATLAB to match their operating frequencies.

## Experimental setup

### Microphone unit placement

Before the start of the experiment in the first session, each subject was asked to form the smallest possible angle between the wrist and the palm of their right arm by the hand as much as possible. This practice enables a distance measurement from the distal bracelet line under which we observed maximum skin displacement (wrinkles) at the wrist with naked eyes. We call this area wrinkle zone as indicated in Fig 1, where the placement of microphone units is avoided to reduce the sensor dislocation during hand motion. Table 1 provides wrinkle zone measurements of the subjects.

**Table 1:** Details of subjects.

Subject	Sex	Age	Wrinkle Zone Length (cm)	Forearm Length (cm)
1	Male	26	3.2	29
2	Female	22	1.8	25
3	Male	29	3.5	26
4	Male	26	3.0	25
5	Female	24	2.0	26
6	Female	23	2.1	27
7	Female	22	2.5	30
8	Male	24	3.3	29
9	Female	27	2.5	23
10	Male	30	2.3	28

In the start of each experimental session, we marked the wrinkle zone length on the wrist. We used this marking for placement of the sensor band at a consistent distance from the hand during all the experiment sessions with each subject. Next, we placed the sensors as indicated in Fig 1. Here, each microphone was placed equidistant from adjacent microphones in the band.

### Data collection

**Fig 3:**
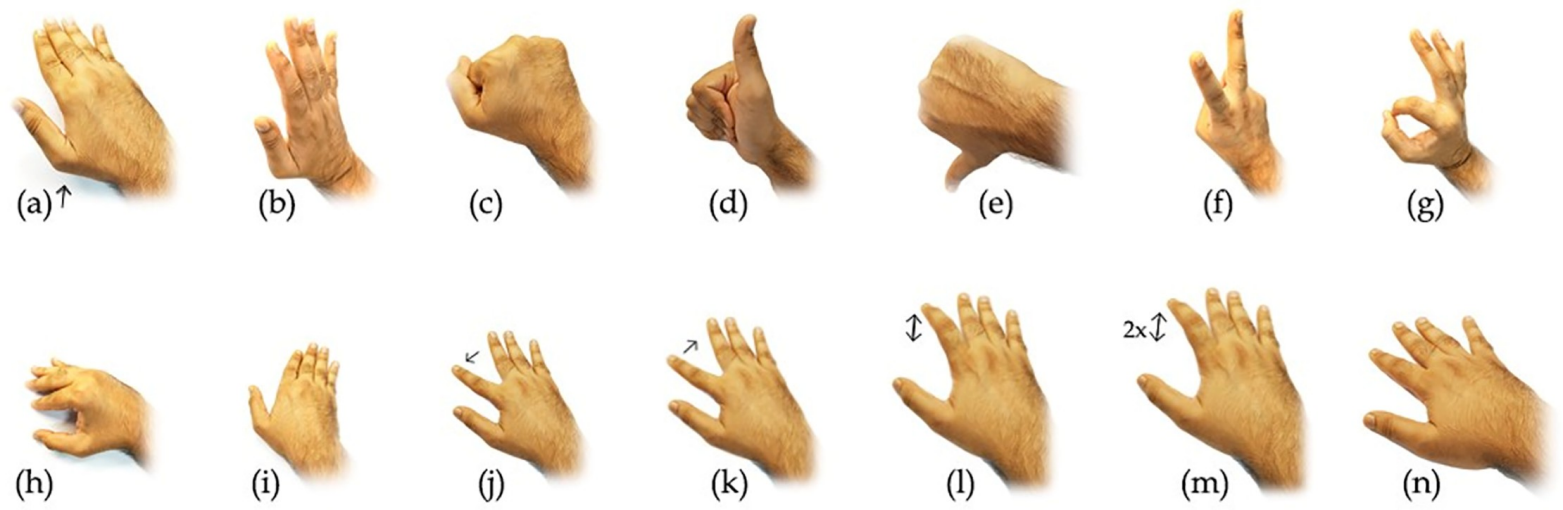
14 hand gestures used in the experiment. (a) Hand lift, (b) hand up, (c) fist, (d) thumbs up, (e) thumbs down, (f) victory, (g) okay sign, (h) hand swipe left, (i) hand swipe right, (j) index finger swipe left, (k) index finger swipe right, (l) index finger single tap, (m) index finger double tap, and (n) relaxed.

Ten right-handed subjects, 5 men and 5 women, participated in the study. Participants provided their informed consent to the experimental procedure, which was approved by the ethics committee at the City University of Hong Kong. The hand gestures under investigation included the 13 common gestures shown in Fig 3, and 1 additional gesture corresponding to the relaxed stage, where the hand is relaxed during the recording, as shown in Fig 3N.

Each subject practiced the gesture before the start of gesture recordings. During the recording session, the subjects were asked to rest their right forearm and elbow on a chair armrest. The position of hand was maintained parallel to the armrest with the palm facing down. They were allowed to make any natural movement of the forearm when they felt it was necessary for the gesture, e.g., in case of thumbs down when there could be a natural twist at the elbow for some of the subjects. Each trial started from the hand in a relaxed position with the palm down and ended at the same resting position. Hence, every channel recording shows the waveform in the action sequence of rest-activity-rest-activity-rest, where the first activity refers to the transformation of hand from the relaxed state to the gesture state and the later activity is back to the relaxed state at the natural pace of the subject.

Each subject performed 10 trials for each gesture. The experiments were conducted in two to three sessions to avoid muscle fatigue, and the duration for each session was no longer than 30 minutes. After sensors placement, an average of 60 trials were recorded in each session. The sensors band was dislodged from the limb at the end of every session and reattached in the subsequent sessions. Therefore, all the 140 trials were recorded from each subject over 2–3 experiment sessions. The data collected allowed us to investigate the reliability of hand gesture recognition across sessions using an acoustic modality.

## Data analysis

### Dataset

The dataset for each subject was a collection of 140 trials (14 gestures × 10 trials), where each trial contained parallel acoustic recordings from 10 microphones, along with motion recordings from the IMU. Each trial spans over three seconds.

### Feature extraction

***M***_***i***_ represents the microphone *i* = 1, 2,…, 10; and *A* and *G* represent the accelerometer and gyroscope with independent values in each specific direction x, y, and z, respectively, for subject *n*. Each trial (*tr*) has 16 independent sensor readings at each time point. A total of 7873 features were extracted using the highly comparative time-series analysis code toolbox in MATLAB [22] from each of these sensor readings, as shown in Eq (1)

$$F={\left[f_{tr,1},f_{tr,2},\cdots ,f_{tr,7873}\right]}_{tr=1,2,\cdots ,140}$$ (1)

where ***F*** is the feature matrix with 140 trials and 7873 calculations per trial of any time-series signal. Using ***F*** for each of the sensors readings, we formed the following cascaded feature matrix:

$$S_{n}=\left[F_{M_{1}},F_{M_{2}},\cdots ,F_{M_{10}},\;F_{A_{x}},F_{A_{y}},F_{A_{z}},\;F_{G_{x}},F_{G_{y}},F_{G_{z}}\right]$$ (2)

where ***S***_***n***_ represents the feature matrix of a dimension of 140 × 125,968 (16 signals × 7873 features) for subject *n*. After obtaining ***S***_***n***_, feature scaling was performed using standardization (per feature, per channel) for each subject, so that every feature column of ***S***_***n***_ had a zero mean and unit variance. This can be demonstrated mathematically:

$$S_{n}{}^{\prime }=\frac{S_{n}-\bar{S_{n}}}{\sigma_{n}}$$ (3)

where $\bar{S_{n}}$ and ***σ_n_*** are the mean and standard deviation, respectively, of individual features across all sessions for the subject *n*.

### Feature selection and classification

For the proof-of-concept of the use of multimodal sensors, we employed a generic approach using widely-used feature selection and classification algorithms [23]. Minimum redundancy maximum relevance (mRMR), was applied to the standardized feature matrix obtained from Eq (3). This method ranks the relevance of each feature for classification tasks in large multi-channel, multi-featured datasets. The procedure was adopted from Estevez et al. [24].

After extraction and selection of relevant features, two classifiers were used for classification; a multiclass SVM classifier (using Gaussian kernel) with 10-fold cross-validation, and an LDA classifier with Monte-Carlo cross-validation using 50 runs. In the latter, each run consisted of 112 random training trials (8 trials/gesture) with the remaining 28 trials (2 trials/gesture) acting as the testing set.

## Results

**Fig 4:**
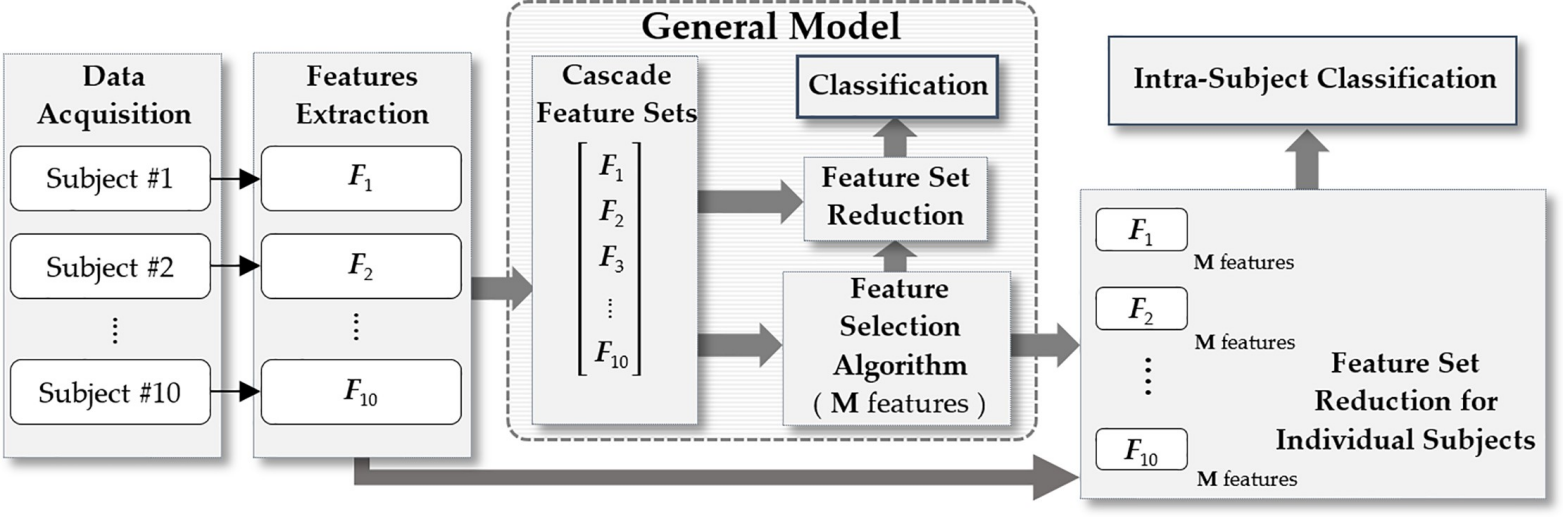
Procedure for data analysis. The first step is feature extraction from individual subjects, then consolidating extracted feature sets from all subjects and applying mRMR to obtain a general model with M features. Then, we applied the same features from the general model on individual subjects to obtain intra-subject classification accuracy.

**Fig 5:**
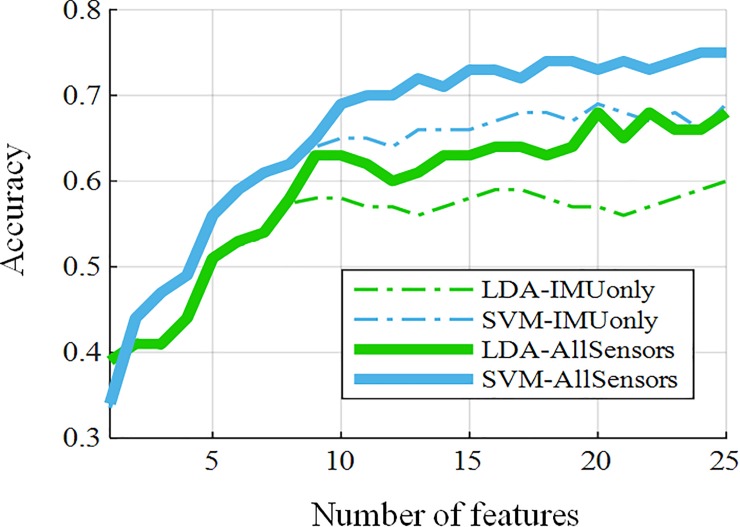
Feature selection with an increasing feature set using concatenated data from all subjects.

The overall analysis was conducted in two parts after feature extraction, which is illustrated in Fig 4. First, we created a general model that includes cascaded standardized feature matrices ***S***_***n***_′ from all subjects, *n* = 1, 2, …, 10. Next, a mutual information-based feature selection algorithm mRMR was applied for feature selection over the cascaded feature set. The top 25 features that resulted from mRMR were noted, which are defined in S1 Appendix, whereas the rankings and locations of the independent signal sources along with feature names are listed in Table 2. The x, y, and z directions of the accelerometer and placement of microphone sensor unit #3 are depicted in in Fig 1. The x, y, and z directions associated with gyroscope represent the angular velocity around the respective directions. Each feature rank corresponds to a feature computation associated with 1 of the 16 independent sources (10 microphones, 3 accelerometers, and 3 gyroscopes). The two features in Table 2, ranked #9 and #23, were selected from the microphone sensor unit at position #3, whereas the rest of the features were selected from IMU data as the result of mRMR. The microphone sensor unit in position #3 indicates the mid-anterior wrist. Later, SVM and LDA classification methods were applied using the 25 selected features in the cascaded feature matrix, and the results are plotted in Fig 5. This resulted in a 75% accuracy of the general model, notably using acoustic information from the mid-anterior wrist and the data from the IMU chip. The same mRMR feature selection analysis with LDA and SVM was also conducted using the data from the IMU only, and the results are also plotted in Fig 5. The comparison shows an improvement of almost 7% using IMU and microphone #3 compared with IMU-only sensing in the general model with basic feature modeling.

**Table 2:** Feature rankings with the independent signal source.

Feature Rank	Feature	Signal Source		
		Accelerometer	Gyroscope	Microphone
1	Shannon Entropy	.	y	.
2	The separation between the maxima of normal distribution and empirical distribution	.	x	.
3	Minimum Bayesian information criterion	.	y	.
4	Mean of autocorrelation of signal segments	.	y	.
5	Standard deviation based stationarity	y	.	.
6	The maximum mean to mean variance by splitting the signal into x segments	.	y	.
7	Kurtosis	z	.	.
8	Shannon Entropy	x	.	.
9	The separation between the maxima of normal distribution and empirical distribution	.	.	3
10	Pearson Skewness	.	x	.
11	Approximate entropy	.	y	.
12	Minimum Bayesian information criterion	x	.	.
13	Mean of autocorrelation of signal segments	z	.	.
14	Permutation Entropy	.	y	.
15	The highlowmu statistic	.	x	.
16	Burstiness	.	y	.
17	The second-order moment	.	z	.
18	The highlowmu statistic	x	.	.
19	The maximum mean to mean variance by splitting the signal into x segments	z	.	.
20	Mean and Standard deviation based stationarity	.	y	.
21	Proportion of data points within ρ standard deviations of the mean	.	x	.
22	Interquartile range	.	y	.
23	Permutation Entropy	.	.	3
24	Mean of 25% (highest and lowest) trimmed signal	x	.	.
25	Kurtosis	y	.	.

**Fig 6:**
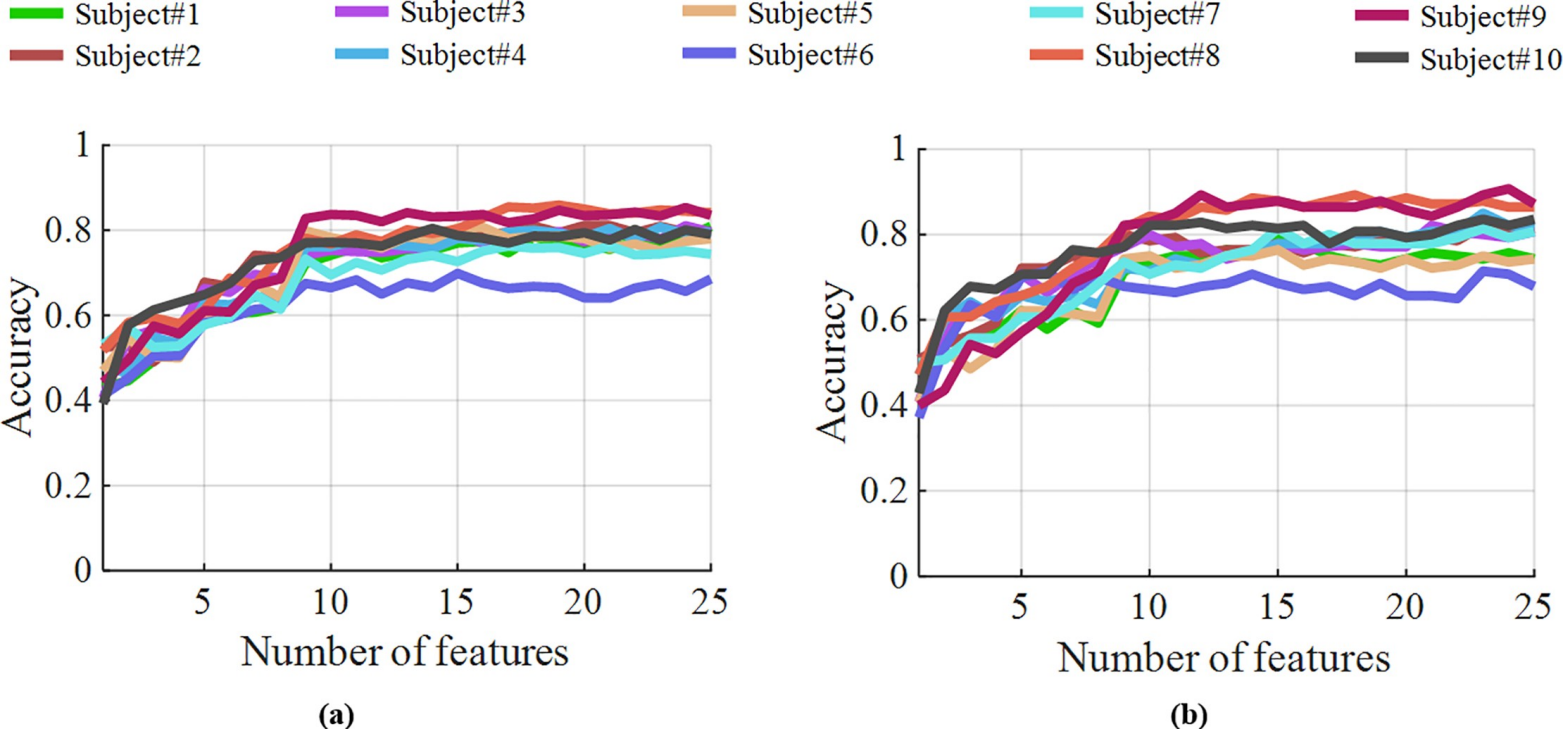
Feature selection with an increasing feature set for each subject separately using the feature set obtained from the global model. (a) classification using LDA and (b) classification using SVM.

**Fig 7:**
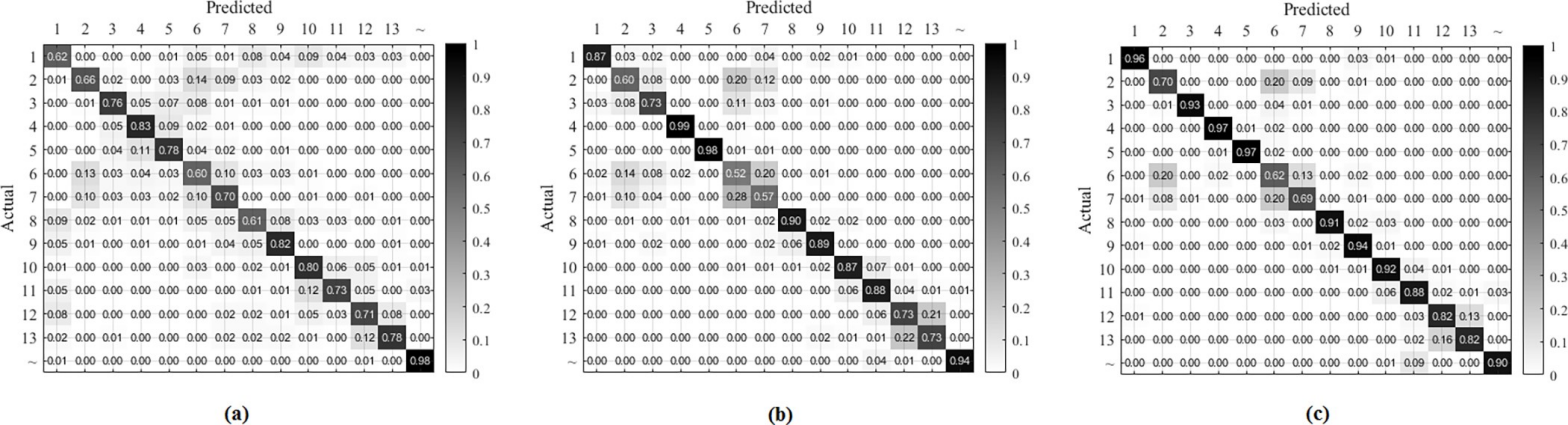
Average confusion matrices using the top 25 feature set obtained from the general model and used for each subject separately with SVM classifier. (a) microphones only; (b) IMU only; (c) all sensors or, more specifically, IMU and Microphone at position #3.

The second part of the analysis was based on using the selected 25 features from the general model for the individual subjects employing the same classification techniques. The results obtained from this analysis are presented in Fig 6, which demonstrates that the features obtained from the general model worked satisfactorily by yielding average accuracy results higher than 80% using the two sensors: the IMU and the microphone placed at position #3. Next, the confusion matrix was formed using the selected features with SVM classification from the individual subjects and averaged across all the subjects, which is shown in Fig 7C. A similar approach was used for the microphones-only and IMU-only cases, whose confusion matrices for comparison are shown in Fig 7A and Fig 7B, respectively. These confusion matrices indicate prominent confusion in some of the gestures that are apparently similar. For example, gestures #2, #6, and #7, which are hand stop, victory, and okay sign, respectively, involve a similar major twist at the wrist. Likewise, no major wrist movement occurs when forming the click/tap and double click/tap gestures i.e., gestures #11 and #12, respectively. Fig 7 indicates that microphones could also enhance hand gesture classification accuracy.

## Discussion

In this study, we explored the feasibility of using a device with an acoustic sensor with an IMU for multimodal hand gesture recognition. Ten microphone sensor units were placed in skin contact around the wrist in the shape of a band. One IMU was attached to the band for recording limb movement during hand gesture. Ten subjects were recruited, and each performed 13 daily life hand gestures (10 trials/gesture) and a relaxed gesture. A total of 7873 features were used to build a feature vector for each recorded trial from 16 independent sources. To create a general model, the top 25 features were selected using a mutual information algorithm (mRMR) by cascading all individual feature matrices from every subject. Next, these selected feature sets from the general model were used for classification in individual subjects. The intra-subject average accuracy was almost 80%. The confusion matrices indicate difficult in distinguishing similar gestures.

From these 25 features, two features were chosen from the recordings of the microphone sensor unit at the mid-anterior wrist, notably at ranks #9 and #23, whereas the rest of the features were selected from the IMU that was attached to the band at the mid-posterior wrist. These results showed that an extra acoustic sensor(s) positioned at mid-anterior wrist could improve hand gesture recognition accuracy of common wrist wearables with an IMU. The information from microphone units could resolve the signal corruption issue faced by IMU during limb movements because pressure-based sensing only records the data when it records the vibrations over the skin due to hand movement at the wrist; therefore, the microphones are virtually free from the major impacts of limb movement. Future improvements in the design of the microphone sensing units could improve sensing and increase the capability of pressure-based sensors in multimodal systems to recognize gestures.

Subjects were tested on the current gesture set for benchmarking purposes, and more hand gestures will be included in future studies. Our current analysis builds a framework for using IMU with acoustic sensing. Currently, the main constraint with using microphones is the inconvenient coupling mechanism with the wrist that uses double-sided adhesive tape to demonstrate how vibrations measured at wrist during finger movements could facilitate hand gesture recognition. Future work will involve the implementation of a neural network to maximize the accuracy obtained with the dual sensors and better coupling mechanism of acoustic sensors at wrist. We also envision these new findings could facilitate the development of electronic tattoo for human-computer interface [25,26]. This will enable the development of a low-cost wrist band with an IMU and an acoustic sensor(s) that can accurately recognize hand gestures in daily life.

## Supporting information

S1 Appendix Selected features and their parameters. (DOCX)
