# Supplementary material for: Multimodal hand gesture recognition using single IMU and acoustic measurements at wrist
Source: PLoS One. 2020 Jan 13;15(1):e0227039. doi: 10.1371/journal.pone.0227039 (PMC6957149; doi:10.1371/journal.pone.0227039)
Supplement: S1 Appendix — (DOCX) [file pone.0227039.s001.docx]

# **S1 Appendix**

## **Selected features and their parameters**

**Table 1. Feature ranking with an independent signal source [1].**

| **Feature Rank** | **Feature** | **Parameters (if any)** |
| --- | --- | --- |
| 1, 8 | Shannon Entropy | - |
| 2, 9 | The separation between the maxima of normal distribution and empirical distribution | - |
| 3, 12 | Minimum Bayesian information criterion | - |
| 4, 13 | Mean of autocorrelation of signal segments | No. of Signal Segments = 3 |
| 5 | Standard deviation based stationarity | Let σ_1_, σ_2_ and σ_3_ represent standard deviations of three equal windows of given signal. Let σ_win_ and σ represent standard deviation in [σ1, σ2, σ3] and the given signal, respectively. The standard deviation based stationarity is then expressed as y = σ_win_/σ |
| 6, 19 | The maximum mean to mean variance by splitting the signal into x segments | No. of Signal Segments = 10 |
| 7, 25 | Kurtosis | - |
| 10 | Pearson Skewness | - |
| 11 | Approximate entropy | Embedding Dimension = 2, Tolerance = 0.2 |
| 14, 23 | Permutation Entropy | Embedding Dimension = 2, Time delay for embedding = 1 |
| 15, 18 | The highlowmu statistic | Let μ_High_ and μ_Low_ be the means of all the data samples in x above μ and below μ, respectively; then highlowmu statistic is given by $y= \frac{\mu_{High}- \mu}{\mu- \mu_{Low}}$ |
| 16 | Burstiness | - |
| 17 | The second-order moment | - |
| 20 | Mean and Standard deviation based stationarity | This algorithm is based on dividing the given time-series signal in four equal windows with μ_1_, μ_2_, and μ_3_ being the means of respective windows. Let σ_win_ and σ represent standard deviation in [μ_1_, μ_2_, μ_3_, μ_4_] and the given signal, respectively. The mean and standard deviation based stationarity is then expressed as y = σ_win_/σ |
| 21 | Proportion of data points within ρ standard deviations of the mean | ρ = 0.3 |
| 22 | Interquartile range | - |
| 24 | Mean of 25% (highest and lowest) trimmed signal | - |

# **Reference**

1. Fulcher BD, Jones NS. hctsa: A Computational Framework for Automated Time-Series Phenotyping Using Massive Feature Extraction. Cell Syst. 2017 22;5(5):527-531.e3.
